# Assuring Healthy Populations During the COVID-19 Pandemic: Recognizing Women's Contributions in Addressing Syndemic Interactions

**DOI:** 10.3389/fpubh.2022.856932

**Published:** 2022-05-27

**Authors:** Rosemary M. Caron, Semra A. Aytur

**Affiliations:** Department of Health Management and Policy and Master of Public Health Program, College of Health and Human Services, University of New Hampshire, Durham, NH, United States

**Keywords:** social determinants of health, exposome, healthy populations, Women in Science, COVID-19, syndemic, systemic racism

## Abstract

A syndemic framework examines disease interactions and the contributions of structural, social, economic, and environmental factors that synergistically interact to contribute to adverse health outcomes. Populations residing in environments with structural susceptibilities experience health disparities and syndemics to a greater extent than their less vulnerable counterparts. The interactions among the social determinants of health (SDoH) and the COVID-19 pandemic have had different results for marginalized populations and have worsened health outcomes for many in this synergistic pandemic. Also, the exposome, the exposure measures for an individual over their lifetime and how those exposures relate to the individual's health, may help to explain why some populations experience more serious cases of COVID-19 compared to other groups. The purpose of this perspective is to: (1) examine the relationship between the syndemic model and the SDoH-exposome; (2) highlight, *via* specific examples, the contributions of female health professionals to SDoH and the COVID-19 syndemic in response to the Women in Science Research Topic, and (3) propose health policy to address syndemic-exposome interactions to help mitigate or prevent public health challenges. By investing in policies that assure health for all populations, the investments could pay dividends in the form of a less severe syndemic next time since we are starting from a place of health and not disease. Lastly, due to the magnification of underlying societal inequities laid bare during the COVID-19 syndemic, we support the expansion of the disease-focused syndemic model to include societal syndemics, such as systemic racism.

## Introduction

A prominent figure in the United States (U.S.) public health system in the late nineteenth and early twentieth centuries was Charles-Edward Amory Winslow (1). Winslow defined public health in 1920 and his description of this civic-oriented, and interdisciplinary field is still in use today:

“Public Health is the science and the art of preventing disease, prolonging life, and promoting physical health and efficiency through organized community efforts for the sanitation of the environment, the control of community infections, the education of the individual in principles of personal hygiene, the organization of medical and nursing service for the early diagnosis and preventive treatment of disease, and the development of the social machinery which will ensure to every individual in the community a standard of living adequate for the maintenance of health; organizing these benefits in such fashion as to enable every citizen to realize his birthright of health and longevity” (2).

From this eloquent definition arises the essential services of public health which align with the assessment, policy development, and assurance functions of public health that are responsible for preventing disease and injury, promoting health and wellbeing, and protecting population health (3). These are core concepts and actions that have helped public health systems at the local, state, national, and international levels manage complex public health challenges, for example, childhood lead poisoning, foodborne outbreaks, obesity, the opioid epidemic, and the COVID-19 pandemic.

Our knowledge and practice of public health has evolved to where we now consider the contributions of the social determinants of health (SDoH) to community health issues. The SDoH are defined as those conditions present in the places we live and spend our time to work, learn, and play (4). Representative examples of SDoH include economic stability (e.g., employment, income, and medical bills); neighborhood and physical environment (e.g., housing, transportation, and walkability); education (e.g., literacy, language, and higher education); food (e.g., access to healthy food choices and hunger); community and social context (e.g., social integration, support systems, and stress); and healthcare system access (e.g., health coverage, provider availability, and quality of care) (5). Approximately 80% of health outcomes are estimated to be due to the SDoH. Specifically, social, and economic factors account for 40% of health outcomes and health behaviors and the physical environment account for 30% and 10% of health outcomes, respectively. Clinical care is estimated to account for 20% of health outcomes in populations. Therefore, the SDoH are non-medical factors that can have a significant and broad effect on health outcomes and quality of life indicators (4, 6). Addressing the SDoH is fundamental to eliminating health disparities, promoting health equity, and improving overall population health outcomes (7).

The SDoH and their contributory role to a population's health status have compounded the extent to which chronic illnesses and communicable diseases have distressed populations. This impact is evident during the current COVID-19 pandemic which began in the U.S. in January 2020 and has disproportionately affected racial and ethnic minorities (8). Specifically, elevated rates of COVID-19 morbidity and mortality have been demonstrated in African American, Native Americans, and LatinX populations compared to their White counterparts. Research suggests that SDoH contribute to these findings (8). For example, these racial and ethnic minorities often experience decreased access to healthcare and living and employment conditions that predispose them to exposure to SARS-CoV-2 (8). Environmental and socio-economic factors and structural racism have contributed to the COVID-19 risk in these vulnerable populations since the social systems in place in U.S. communities often perpetuate practices that promote an inequitable distribution of resources due to multiple levels of racism (e.g., institutional racism, personally-mediated racism, and internalized racism) (9, 10). Comparatively, Blacks have experienced the highest COVID-19 diagnoses rates in the United Kingdom (U.K.) and ethnic minorities (specifically Asian populations) have had an elevated mortality rate compared to their White British counterparts (11). In both the U.S. and the U.K, the presence of pre-existing comorbidities (e.g., South Asian populations experience high rates of diabetes and cardiovascular disease; Blacks experience high rates of hypertension) and socio-economic and geographical factors, population density, housing quality, and occupation may account for the observed health disparities during the COVID-19 pandemic (9, 12).

These interactions among the SDoH have played a role in the COVID-19 pandemic in different ways for marginalized populations and have worsened health outcomes for many in this synergistic pandemic, called a syndemic (13). The purpose of this perspective is to: (1) examine the relationship between the syndemic model and the SDoH-exposome; (2) highlight, *via* specific examples, the contributions of female health professionals to SDoH and the COVID-19 syndemic in response to the Women in Science Research Topic, and (3) propose health policy to address syndemic-exposome interactions to help mitigate or prevent public health challenges.

## Syndemic Interactions

### Syndemic Defined

The synergistic interactions among disease and the contextual social environment (i.e., SDoH) in which they occur can result in a syndemic. More specifically, a syndemic is described by the coming together of two or more health conditions (e.g., chronic illness and infectious disease) and their adverse interactions with each other and the SDoH that can provide the milieu for enhancing vulnerabilities, magnifying inequities, and worsening health outcomes for marginalized populations (14–16). A syndemic framework examines disease interactions and the contributions of structural, social, economic, and environmental factors that synergistically interact to contribute to adverse health outcomes for individuals and whole populations (16, 17). Singer first introduced the term *syndemic* in the 1990s when he described the interactions among substance abuse, violence, and AIDS (SAVA syndemic) (15). Singer identified the criteria necessary for a syndemic: (1) grouping of two or more health conditions or diseases in a population; (2) social and contextual factors that allow for the biological interactions to occur and for the development of adverse health outcomes, and (3) disease interactions that lead to negative outcomes (e.g., health, social, and behavioral) for the affected population and increases their health burden (15, 16). Other syndemics identified and studied by women researchers include the HIV, malnutrition, food insecurity syndemic and the violence, immigration, depression, type-2-diabetes, abuse syndemic (VIDDA syndemic) among Mexican women immigrants (15, 16). Syndemics are not restricted to communicable and chronic diseases and may be heightened by health inequality attributable to poverty, structural violence, and stigmatization (16). For example, research has demonstrated that children who encounter adverse experiences during childhood, such as living in a violent neighborhood or witnessing violence in their living environment are three times more likely as children who do not experience similar violence to be diagnosed with asthma. This synergistic relationship of living with fear, violence, and asthma are representative of a syndemic often observed in urban environments (16, 18).

### Literature Review

To respond to the Women in Science Research Topic, we examined the contributions of female researchers to the COVID-19 syndemic. We examined GALE Biography, Historical Abstracts, and PubMed databases. The historical databases were reviewed with an emphasis on the time period: 1800-1918; 1919-1950 (WW1-WW2); and Post-WWII (1951-present). Two U.S. government reports featuring women and minority scientists were also utilized (19, 20). Searches were conducted from November 2021–January 2022.

For the GALE Biography and Historical Abstracts databases, we used the following search strings: [“public health” OR epidemiolog^*^) AND (pioneer^*^ OR famous)]; and [“syndemic” AND (“women” OR “female”) AND (pioneer^*^ OR famous)], which identified 111 citations. We excluded citations if they did not focus: (a) on women's contributions; (b) on public health; or (c) on an English translation. Duplicate citations were excluded. A total of 23 citations resulted, highlighting women's significant contributions to public health in clinical, research, and policy/advocacy domains from the pre-Industrial Revolution period through the Post WW-II period.

For the PubMed database, using the search phrase, “syndemic and public health and female authors”, 69 articles were identified with 51 including the term “syndemic” in the abstract, title, or narrative of the article. Using the search phrase, “COVID-19 syndemic and public health and female authors”, 7 of the 51 published articles were identified. The articles in the PubMed database identified the long-standing SAVA syndemic and more recently the COVID-19 syndemic with the range of COVID-19 syndemic articles published between 2020 and 2021.

### Women Health Professionals' Contributions to the SDoH and Syndemics

The historic literature review identified female health professionals' “pre-syndemic” contributions, which are often unrecognized in public health textbooks and curricula. The authors, who are both female, further note that this work provided a strong foundation upon which the essential public services of contemporary public health could be built. For example, female health professionals advanced the fields of public health nursing across the globe, contributed to our understanding of the zoonotic transmission that foreshadowed the contemporary One Health model, campaigned for gender-sensitive policies, established community clinics focusing on the overall wellness of the community, and expanded our scientific knowledge through research in fields such as bacteriology and immunology. The work of these women foreshadowed the need to address syndemics *via* a SDoH perspective.

The relationship between the SDoH and the COVID-19 syndemic is also evident in the results from the literature search which identified the following select global contributions from women health professionals:

- Active dengue (endemic vector-borne disease) infection and co-infection with SARS-CoV-2 in Brazil resulted in severe pulmonary conditions and contributed to burdening an overwhelmed healthcare system and warranting preventive measures (21); the syndemic nature of dengue and COVID-19 warrants a promotion of preventive measures and further investigation into their epidemiological and clinical interactions in Peru (22).
- Mentoring as a solution to develop women and underrepresented racial and ethnic minorities as faculty and leaders in U.S. academic medicine settings during the racism and COVID-19 syndemics (23).
- Preventive measures to restrict physical contact during the COVID-19 syndemic has affected healthy nutrition practices (24).
- The racism and COVID-19 syndemics and their associated vulnerabilities need to be considered for Black women and pregnant women in the U.S. Prevention approaches should consider SDoH that contribute to the grouping of vulnerabilities (25).
- Essential workers who reside in middle-income countries (i.e., Brazil and Spain) with elevated rates of inequality may face challenges during the COVID-19 syndemic (e.g., mental health support) due to COVID-19 and SDoH interactions (26).
- A syndemic framework should be applied to populations co-infected with COVID-19 and HIV in Nigeria. Also, there is a need to strengthen healthcare delivery services in response to the current public health challenges (27).

Based on the work described above and prior research in this area, populations residing in environments with structural susceptibilities experience health adversities and syndemics to a greater extent than their less vulnerable counterparts which warrants a review of the contributory upstream determinants (e.g., social, economic, and political factors) (17).

Women researchers have proposed that the interactions between the COVID-19 syndemic and the SDoH engage in a bi-directional relationship (28). In this relationship, structural determinants (e.g., poverty, and violence); sociocultural determinants (e.g., lifestyle, family conflict, and support); socioeconomic determinants (e.g., employment and access to health care); and individual determinants (e.g., health behavior and self-efficacy) interact with each other. For example, if one is employed in a service industry as an hourly employee then they probably need to keep going to work due to the lack of sick time. If they do not work, they will not be paid which means they can't provide food and shelter for themselves or families. As a result, many of these people put themselves at risk of exposure to SARS-CoV-2 because the interactions of the SDoH with the COVID-19 syndemic removes their choice to prioritize their health (15, 17).

To assure the health of populations experiencing these syndemics, the authors propose, based on female health professionals' contributions in this area, that significant reform of public health policy in the U.S. occurs that assures a basic standard of living where housing is affordable, a minimum wage allows for healthy food choices, and universal healthcare, as a few starting points. Such systemic change will take years but if the COVID-19 syndemic has taught us anything, it is that to survive the next pandemic, with the least amount of morbidity and mortality, will require starting with a healthy population and not the magnitude of widespread inequity that currently exists in the U.S.

Supplementary Table 1 highlights the myriad contributions of female health professionals to the pre-syndemic and COVID-19 syndemic domains.

### Syndemics and the Exposome

Another area that warrants attention is the role of the exposome in syndemics. The exposome, coined by Wild in 2005 (29) is intended to complement the genome, and is defined as the exposure measures for an individual over their lifetime and how those exposures relate to the individual's health (30, 31). Exposures can include determinants in the form of the natural environment (e.g., air pollution and toxicants), built environment (e.g., greenspace and walkability) and social environment (e.g., neighborhood deprivation and lifestyle) (32, 33). These interactions have been shown to have negative effects on asthma, cardiovascular disease, diabetes, and hypertension which have been reported as co-morbidities for severe COVID-19 (32, 34–36).

The exposome may help to explain why some populations experience more serious cases of COVID-19 compared to other groups (37). Studies have shown that SARS-CoV-2 binds to a human angiotensin converting enzyme 2 (ACE2) receptor located on the epithelial cells of the lungs, blood vessels, brain, kidney, and intestines (38). Exposure to environmental toxins (e.g., air pollution), lifestyle behaviors (e.g., high fat diet), and underlying disease (e.g., diabetes) can affect the expression of ACE2 and possibly increase the severity of the SARS-CoV-2 infection (37). Therefore, it is possible that gene and environmental interactions could account for varying rates of COVID-19 susceptibility among different racial and ethnic groups (37–39).

Female scientists are also contributing directly to exposome research. For example, the Hercules project at Emory University was funded by the U.S. National Institute of Environmental Health Sciences in 2013 to support exposome research. Several women participated in discovering, implementing, and evaluating the application of the exposome concept to advance our understanding of how gene-environment interactions affect population health (40). For example, women researchers have also been studying how certain air pollutants may affect the brain and increase the risk for dementia. The study combines survey data with traffic-related air pollution data based on geographic location, enabling researchers to evaluate relationships between air pollutant mixtures and stages of cognitive decline.

Another female researcher has been developing a novel model to assess the effects of metal exposure on fetal lung development. The research examines whether pregnant women's exposure to heavy metals, such as cadmium and arsenic, interferes with fetal lung development. This study advances our understanding of how environmental exposures that occur prior to birth may contribute to pulmonary disease. Additional women researchers are examining the intersection of genomics, cultural competency, and health disparities, calling for the establishment of culturally competent systems of care (41).

**Figure 1:**
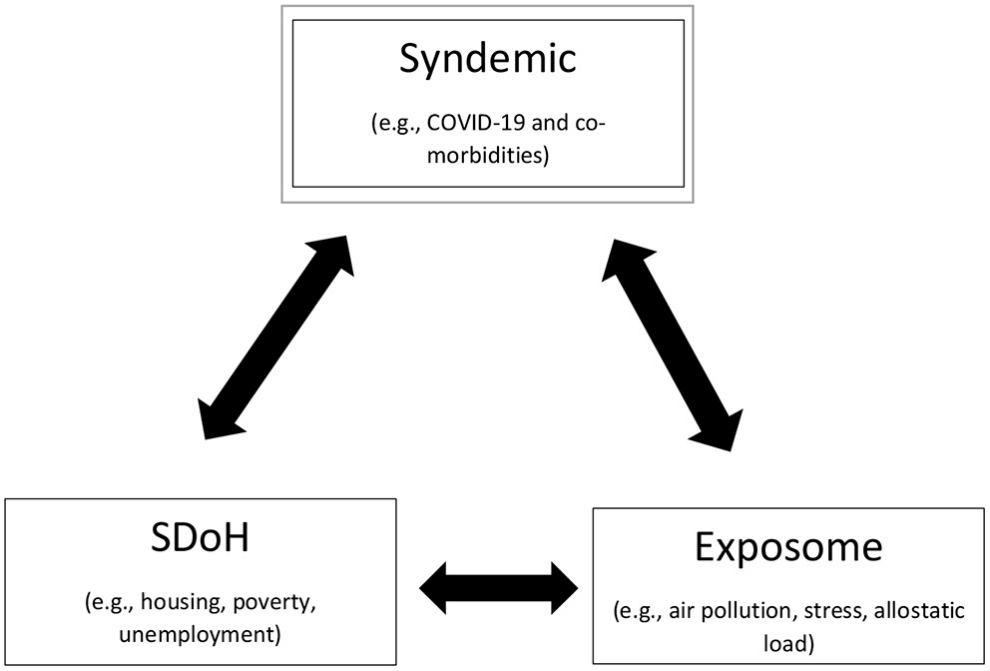
Syndemic interactions among the SDoH-exposome. We propose that in addition to a bi-directional relationship between SDoH and a syndemic, that there is potential for a bidirectional relationship between the exposome and a syndemic and a bi-directional relationship between the exposome and SDoH. For example, populations residing in overcrowded, poor quality housing that is zoned between an auto-body shop and a crematorium in an urban environment may be living with historical housing segregation practices. This population may work in a service occupation earning an hourly wage, may be exposed to air pollutants and toxicants *via* their living environment and the public transportation system that is necessary to attend work, and run errands. The local food source is a centralized convenient store which does not provide a reliable supply of fruits and vegetables at an affordable price. This population resides in a high crime neighborhood where the murder rate, gang violence, and illegal drug use are increasing. These SDOH-exposome interactions make one more susceptible to the COVID-19 syndemic due to the multitude of exposures encountered. In turn, these conditions contribute to a syndemic hotspot of COVID-19, substance misuse, and toxic stress accelerating negative environmental exposures (e.g., medical waste, hypodermic needles, increased carbon emissions and worsening air quality) that act as force multipliers for the population's allostatic load.

Building on the work of female health professionals who have preceded them, the authors of the present study propose that in addition to a bi-directional relationship between SDoH and a syndemic, that there is potential for a bi-directional relationship between the exposome and a syndemic and a bi-directional relationship between the exposome and SDoH (Figure 1). For example, populations residing in overcrowded, poor quality housing that is zoned between an auto-body shop and a crematorium in an urban environment may be living with historical housing segregation practices. This population may work in a service occupation earning an hourly wage, may be exposed to air pollutants and toxicants *via* their living environment and the public transportation system that is necessary to attend work, and run errands. The local food source is a centralized convenience store which does not provide a reliable supply of fruits and vegetables at an affordable price. This population resides in a high crime neighborhood where the murder rate, gang violence, and illegal drug use are increasing. These SDOH-exposome interactions make one more susceptible to the COVID-19 syndemic due to the multitude of exposures encountered. In turn, these conditions contribute to an evolving syndemic hotspot of COVID-19, substance misuse, and toxic stress accelerating negative environmental exposures (e.g., medical waste, hypodermic needles, and increased carbon emissions) that act as force multipliers for the population's allostatic load (42).

The environment has been reported to be responsible for ~70–90% of chronic disease while one's genome is purported to be responsible for 10–30% of chronic disease (43). Thus, a review of the exposome may complement a syndemic mitigation approach that considers the SDoH, political, and economic factors that propagate biological and societal epidemics.

## Discussion

C-E.A. Winslow's public health definition is still applicable today when addressing complex public health challenges. The COVID-19 pandemic, arguably one of the most complex public health challenges of the twenty-first century to date, has been described as a syndemic that is compounded by pre-existing underlying disease and the SDOH-exposome. These syndemic interactions may worsen the morbidity and mortality for marginalized populations but these relationships could also direct our limited resources to mitigating or preventing adverse health effects for future public health challenges. For example, there is a role for policy, or as Winslow calls the “social machinery” (1), to improve the conditions in which people live, work, and play. Policy interventions could assure healthy populations by providing equitable access to quality healthcare, affordable and safe housing, guaranteed minimum living wage, and healthy and affordable food options. By investing in policies that assure health for all populations, the investments could pay dividends in the form of a less severe syndemic next time since we are starting from a place of health and not disease. Recognizing women's contributions to population health and developing policies that support pathways of opportunity for women to assume leadership roles in institutions such as government, higher education, and healthcare will enable us to work toward favorable SDoH-exposome interactions that may reduce the burden of future syndemics. Examples of such policies include paid maternity/paternity leave, childcare, elder-care support, access to safe reproductive healthcare services, living-wage policies, and workplace policies ensuring safety and work-life balance (44).

The COVID-19 syndemic has made visible underlying, pervasive systemic flaws, such as structural racism, in the education, housing, and employment sectors, and has forced public health to acknowledge racism as a crisis. A female researcher calls for, and the authors agree, that the concept of syndemics needs to move beyond disease-oriented syndemics and expand to include societal epidemics: “that social phenomena such as direct violence (e.g., interpersonal violence, genocide, ethnic cleansing, colonialism, and imperialism) and structural violence (e.g., poverty, racism, historical trauma, and political disenfranchisement) are widespread and adversely affect health in many…communities, thus meeting the definition of an epidemic” (45).

Structural or institutional violence, specifically systemic racism, refers to practices that are embedded in local, state, and federal policies and provide an advantage to certain populations and not others (10, 46). The interventions to address systemic racism require structural interventions *via* policy reform across public and private sectors including, but not limited to, health, education, employment, and the criminal justice system (46). The development, implementation, and assessment of equitable economic policies that address poor quality housing, unemployment, poverty, and racial segregation could help to reduce socio-economic inequalities among racial and ethnic minorities and help to minimize the burden of disease (46, 47).

The bi-directional relationship among the SDoH, including the political and economic determinants, exposome and a syndemic, whether biological or societal in nature, can be synergistic at a population level and have widespread consequences (48). The spread of a disease or selective behaviors in an epidemic or pandemic “always follow the fault lines of society” (49) and depending on the syndemic interactions, the inequities may become visible or magnified despite always having been present as has occurred during the COVID-19 syndemic.

To serve as a call to action to address syndemic interactions and assure healthy populations in the face of complex public health challenges, we have highlighted work conducted globally by female healthcare professionals that have informed our pre-syndemic environment and the current COVID-19 syndemic. As a result of this work, we call for transformative changes across governments, systems, and infrastructures to support gender-sensitive policies that will improve the SDoH-exposome interactions as key drivers of health (50). As the COVID-19 syndemic has demonstrated, *via* the contributions of many women researchers, all populations are interconnected, and addressing the interactions that contribute to preventable morbidity and mortality will facilitate reductions in the global burden of disease for all people.

## Data Availability Statement

The original contributions presented in the study are included in the article/supplementary material, further inquiries can be directed to the corresponding author/s.

## Author Contributions

RC proposed the topic for discussion and took the lead in writing the article. RC and SA co-developed the manuscript outline. All authors contributed to the article and approved the submitted version.

## Conflict of Interest

The authors declare that the research was conducted in the absence of any commercial or financial relationships that could be construed as a potential conflict of interest.

## Publisher's Note

All claims expressed in this article are solely those of the authors and do not necessarily represent those of their affiliated organizations, or those of the publisher, the editors and the reviewers. Any product that may be evaluated in this article, or claim that may be made by its manufacturer, is not guaranteed or endorsed by the publisher.
