# Supplementary material for: Assuring Healthy Populations During the COVID-19 Pandemic: Recognizing Women's Contributions in Addressing Syndemic Interactions
Source: Front Public Health. 2022 May 27;10:856932. doi: 10.3389/fpubh.2022.856932 (PMC9197070; doi:10.3389/fpubh.2022.856932)
Supplement: Supplementary file 1 [file Table_1.docx]

**Table 1. GALE Biographies and Historical Abstracts identified with the search strings: [“public health" OR epidemiolog*) AND (pioneer* OR famous)]; and [“syndemic” AND (“women” OR “female”) AND (pioneer* OR famous)]; additional sources included the U.S. Centers for Disease Control and Prevention and the National Park Service.**

| **Pre-Syndemic Contribution** | **Domain** | **Date** | **Country and General Focus of Study** | **Citation** |
| --- | --- | --- | --- | --- |
| 1. African auxiliary health personnel were trained to work within the Western biomedical system in South Africa in the early 20th century. These healthcare workers played  pivotal roles ranging from chronic disease management to violence prevention to vaccine dissemination; foreshadowing the need to address synergistic epidemics through a syndemic perspective. | Clinical Care | 1900-1950 | Africa  Assistant health workers;  medical translation and vaccine dissemination | Noble, V. 2006. “Health is much too important a subject to be left to doctors.” African assistant health workers in natal during the early twentieth century. Journal of Natal & Zulu History; 25: 95-134. doi.org/10.1080/02590123.2006.11964138 |
|  |  |  |  |  |
| 2. Sara Bodek Paltiel was a Jewish-American health professional whose work advanced the role of Jewish women in public health nursing in Israel and Palestine.  Her work helped to advance our understanding of how social and economic inequality shape health outcomes. | Clinical care | 1932-1993 | Israel and Palestine  Public health nursing | Rosenfeld, P. 2001. From the lower east side to the upper Galilee: The pioneering experiences of Sara Bodek Paltiel. Nursing History Review; 9: 141-158. |
|  |  |  |  |  |
| 3. Anna Hellebrandt was a female researcher who earned degrees in anatomy and physiology before leaving the United States to study at the Clinic for Sportsmen in Prague (now capital of the Czech Republic). At the beginning of  World War II, she returned to the United States to specialize in physical rehabilitation. | Academic research | 1901–1992 | United States; Czech Republic  Research on anatomy, physiology, and physical rehabilitation | Wrynn AM. 1999. Frances Anna Hellebrandt: physician, mentor, and pioneer in exercise physiology. Res Q Exerc Sport; ;70(4):324-34. doi:10.1080/02701367.1999.10608053.  PMID: 10797891. |
|  |  |  |  |  |
| 4. Helena Kagan was a pediatrician and social activist for peace in Jerusalem. She was instrumental in expanding access to health care in Israel. She devoted her life to improving welfare services and living conditions for children of all religious affiliations. | Clinical care | 1889- 1978 | Israel | Rubin, Z. 2008. Helena Kagan (1889–1978): the first paediatrician in Israel. Journal of Medical Biography;16(3):144-149. doi:10.1258/jmb.2007.00704 |
|  |  |  |  |  |
| 5. Josephine Elizabeth Butler was a British political activist and social reformer who campaigned for the right of women to quality education, the abolition of child prostitution, and an end to human trafficking  of young women and children. | Governmental public health;  political activist | 1828 –1906 | Britain  Social reform; maternal/child health | Boyd, N. 1982. Three Victorian Women Who Changed Their World: Josephine Butler, Octavia Hill, Florence Nightingale. Oxford: Oxford University Press. [ISBN](https://en.wikipedia.org/wiki/ISBN_(identifier)) [978-0-333-30057-2](https://en.wikipedia.org/wiki/Special:BookSources/978-0-333-30057-2). |
|  |  |  |  |  |
| 6. Sara-Zofia Syrkin-Binsztejnowa was a leader in conducting emergency medical relief work and public health work in Poland and the Warsaw Ghetto. | Clinical care | 1891-1943 | Poland  Emergency medical care | Golden, J.D. 2018. “Show that you are really alive”: Sara-Zofia Syrkin-Binsztejnowa’s Emergency Medical Relief and Public Health Work in Early Interwar Poland and the Warsaw Ghetto. Medizinhistorisches Journal, 53(2), 125–162. http://www.jstor.org/stable/44985751 |
|  |  |  |  |  |
| 7. Amalija Simec was a physician and founder of Slovenian epidemiology and bacteriology | Clinical care; research | 1893-1960 | Slovenia  Epidemiology and bacteriology | Toplak, C. 2002.The first Slovenian female scientists: educated Slovenian women from the Middle Ages to the first half of the 20th century. History of Natural Sciences; 15-16:197-210. PMID: 17225365. |
|  |  |  |  |  |
| 8. Dorothy Crowfoot Hodgkin was a pioneering researcher in chemistry.  Her work on the use of X-ray crystallography enabled the development of penicillin and insulin, supporting the management of both infectious and chronic disease.  She won the Nobel Prize in chemistry in 1964. | Academic research | 1910-1994 | Britain, Egypt, Sudan  Chemistry, drug development | Dubb, A. 2002. Women in medicine and science: Dorothy Hodgkin (1910-1994). Adler Mus Bull;28(2-3):17-9. PMID: 20329354. |
|  |  |  |  |  |
| 9. Epidemiologist Anne Hardy’s research documents the historical role of veterinarians in public health in England. There was growing about the role of zoonotic transmission of disease. The exploration of the role of veterinarians in the urban animal economy foreshadows the modern One Health model. | Academic research | 1850-1900 | Britain  Environmental Health; Veterinary public health; One Health | Hardy A. 2002. Pioneers in the Victorian provinces: veterinarians, public health, and the urban animal economy. Urban History;29(3):372-87. doi: 10.1017/s0963926802003036. PMID: 21033524. |
|  |  |  |  |  |
| 10. Lydia Ernestine Becker served as a leader in the early British women’s suffrage movement. She founded the [*Women's Suffrage Journal*](https://en.wikipedia.org/wiki/Women%27s_Suffrage_Journal) between 1870 and 1890. She was also trained as a biologist, and her research in botany  contributed to work that was later published by Charles Darwin. | Governmental public health;  political activist; biological research | 1827-1890 | Britain  Women’s rights; social advocacy; biology | Blackhorn, H. 2013. Women's suffrage: a record of the women's suffrage movement in the British Isles, with biographical sketches of Miss Becker. Charleston: Nabu Press, 2013. [ISBN](https://en.wikipedia.org/wiki/ISBN_(identifier)) [978-1295309993](https://en.wikipedia.org/wiki/Special:BookSources/978-1295309993) |
|  |  |  |  |  |
| 11. Female public health professionals played key roles in the Los Angeles Plaza Community Center (PCC), one of the first community centers that combined a walk-in medical clinic with efforts to address the overall health of the community. The PCC provided multi-faceted social support including assisting with education, nutrition, employment, and transportation. Thus, it became a model for addressing the Social Determinants of Health (SDoH). | Clinical care | 1913-1925 | United States  Social Determinants of Health | Satya-Murti, S., Gutierrez, J. 2019.Addressing the Social Determinants of Health: A Los Angeles Community Center’s Narrative from 1913 to 1925  Southern California Quarterly; 101 (4): 357–395.  <https://doi.org/10.1525/scq.2019.101.4.357> |
|  |  |  |  |  |
| 12. Katherine B. Higgins played a pivotal role in providing social services to support public health in Los Angeles. Higgins opened the first Goodwill store in Los Angeles 1918, in conjunction with the Plaza Community Center (PCC). Goodwill also provided employment, job training, social services to Latin Americans and other immigrants living in Los Angeles. | Clinical care | 1880-1967 | United States  Social Determinants of Health | Los Angeles United Methodist Museum of Social Justice. 2021. Goodwill: Its founding and history in southern California.https://www.museumofsocialjustice.org/goodwill-its-founding-and-history-in-southern-california.html |
|  |  |  |  |  |
| 13. Annie Dodge Wauneka was a public health activist who led efforts to improve the health and welfare of her Navajo people. She wrote a Navajo-to-English medical dictionary and delivered radio broadcasts to inform health literacy and communication. She received the Presidential Medal of Freedom for her efforts in 1963. | Clinical care; advocacy | 1910-1997 | United States  Communicable disease prevention (influenza, trachoma, tuberculosis); environmental health (water contamination), and alcoholism. | National Park Service. (2021. Women in Public Health and Medicine. Accessed 8 January 2022. <https://www.nps.gov/subjects/womenshistory/women-in-health.htm> |
|  |  |  |  |  |
| 14. “The Lakota Grandmothers” - Phoebe Downing (Standing Rock), Eunice Larrabee (Cheyenne River), Alfreda Janis Bergin (Pine Ridge), and Irene Groneau (Sisseton-Wahpeton) worked to improve the health of their tribal communities by addressing multiple health issues including tuberculosis, mental health, and alcoholism. Their efforts contributed to the development of the Community Health Representative programs under the Indian Health Service. | Clinical care | 1953-1970 | United States  Infectious disease; mental health | Centers for Disease Control and Prevention. 2021. American Indian & Alaska Native Contributions to Public Health. Accessed 8 January 2022. <https://www.cdc.gov/tribal/tribes-organizations-health/contributions/index.html> |
|  |  |  |  |  |
| 15. Susan La Flesche Picotte was the first female Native American physician. She was born on the Omaha Indian Reservation and became an important public health activist, promoting hygiene and sanitation measures on the Reservation. | Clinical care; advocacy | 1865- 1915 | United States  Infectious and chronic disease prevention and treatment | Centers for Disease Control and Prevention. 2021. American Indian & Alaska Native Contributions to Public Health. Accessed 8 January 2022. <https://www.cdc.gov/tribal/tribes-organizations-health/contributions/index.html> |
|  |  |  |  |  |
| 16. Susie Walking Bear Yellowtail became the first registered nurse working on the among the Crow Reservation.As a consultant for the US Public Health Service, she fought against the sterilization of Native American women without informed consent and led efforts to establish the Community Health Representative program. | Clinical care; advocacy | 1903-1981 | United States  Public health nursing; infectious and chronic disease | Centers for Disease Control and Prevention. 2021. American Indian & Alaska Native Contributions to Public Health. Accessed 8 January 2022. <https://www.cdc.gov/tribal/tribes-organizations-health/contributions/index.html> |
|  |  |  |  |  |
| 17. Patricia Nez Henderson was a public health researcher who was committed to s committed to improving the health of American Indians. She was the first American Indian woman to graduate from Yale University School of Medicine and was honored with the inaugural Patricia Nez Award. Her research examined the effects of nicotine on Native Americans, and she contributed to the development of culturally-sensitive smoking cessation tools. | Research | 1965- | United States  Chronic disease prevention; smoking cessation | Centers for Disease Control and Prevention. 2021. American Indian & Alaska Native Contributions to Public Health. Accessed 8 January 2022. <https://www.cdc.gov/tribal/tribes-organizations-health/contributions/index.html> |
|  |  |  |  |  |
| 18. Virginia M. Alexander was a pioneering Black physician and public health researcher. Through her studies of racism in the healthcare system, Dr. Alexander identified associations between segregation Black Americans' health. | Clinical care; research | 1900-1949 | United States  Health disparities research | National Park Service. 2021. Women in Public Health and Medicine. Accessed 8 January 2022. <https://www.nps.gov/subjects/womenshistory/women-in-health.htm> |
| 19. Cora Reynolds Anderson was the  first female public health professional to serve in a U.S. state legislature. As a member of the Ojibwa tribe in Michigan (MI), she organized the first public health service in Baraga County, MI, bringing the first public health nurse to the region. She worked to address alcoholism and tuberculosis and led the fight to recognize Native American fishing rights. | Government | 1882-1950 | United States  Government; public health education | National Park Service. 2021. Women in Public Health and Medicine. Accessed 8 January 2022. <https://www.nps.gov/subjects/womenshistory/women-in-health.htm> |
|  |  |  |  |  |
| 20. Marie Equi worked in clinical care for more than 20 years, primarily in maternal child health and reproductive justice. Dr. Equi was an advocate for family planning, labor reform, women’s suffrage, and fair wages. | Clinical care; political activist | 1872-1952 | United States  Maternal child health; reproductive justice; political advocacy | National Park Service. 2021. Women in Public Health and Medicine. Accessed 8 January 2022. <https://www.nps.gov/subjects/womenshistory/women-in-health.htm> |
|  |  |  |  |  |
| 21. Margaret Chung was the first Chinese American female physician who cared for thousands of American service-persons during WWII. She founded one of the first Western medical clinics in San Francisco’s Chinatown. | Clinical care; advocacy | 1889-1959 | United States  Military medicine | National Park Service. 2021. Women in Public Health and Medicine. Accessed 8 January 2022. <https://www.nps.gov/subjects/womenshistory/women-in-health.htm> |
|  |  |  |  |  |
| 22. Alice Hamilton was a physician and a pioneer in occupational and environmental health. She was known for treating working-class immigrant patients  in Chicago. She fought to eliminate health disparities. In 1919, Dr. Hamilton became the first woman on the faculty at Harvard University. | Clinical care; advocacy; academic research | 1869-1970 | United States  Environmental health, toxic chemicals, workplace injuries; infectious disease | National Park Service. 2021. Women in Public Health and Medicine. Accessed 8 January 2022. <https://www.nps.gov/subjects/womenshistory/women-in-health.htm> |
|  |  |  |  |  |
| 23. Helen Rodríguez Trías was the [first Latina director](https://www.nps.gov/articles/latinothemescience.htm) of the American Public Health Association. She was a pediatrician, public health expert and women’s rights activist who advanced our understanding of the social determinants of health.  She also established Puerto Rico’s first infant health clinic. | Clinical care; advocacy; academic research | 1929-2001 | United States; Puerto Rico | National Park Service. 2021. Women in Public Health and Medicine. Accessed 8 January 2022. <https://www.nps.gov/subjects/womenshistory/women-in-health.htm> |

**Table 1. U.S. National Library of Medicine PubMed.gov. articles with the search terms: “COVID-19 syndemic and public health and female authors”.**

| **Syndemic Contribution** | **Domain** | **Date** | **Country and General Focus of Study** | **Citation** |
| --- | --- | --- | --- | --- |
| 1. Data from the COVID-19 syndemic in Brazil demonstrates co-infection of SARS-CoV-2 and dengue affects a significant percentage of COVID-19 patients requiring greater attention and resources from healthcare providers. | Academia, Hospital | 2021 | Brazil  COVID-19 and dengue fever | Teotônio IMSN, de Carvalho JL, Castro LC, Nitz N, Hagström L, Rios GG, de Fátima Rodrigues de Oliveira M, Dallago BSL, Hecht M. Clinical and biochemical parameters of COVID-19 patients with prior or active dengue fever. Acta Trop. 2021 Feb;214:105782. doi: 10.1016/j.actatropica.2020.105782. Epub 2020 Nov 28. PMID: 33259817; PMCID: PMC7698682. |
|  |  |  |  |  |
| 1. Role that mentoring can play for women and people of color, especially when working in institutions that lack diversity and managed the racism and COVID-19 syndemic. | Academia, Professional Association | 2021 | United States  Racism and COVID-19 | South-Paul JE, Campbell KM, Poll-Hunter N, Murrell AJ. Mentoring as a Buffer for the Syndemic Impact of Racism and COVID-19 among Diverse Faculty within Academic Medicine. Int J Environ Res Public Health. 2021 May 5;18(9):4921. doi: 10.3390/ijerph18094921. PMID: 34063085; PMCID: PMC8125270. |
|  |  |  |  |  |
| 1. Systematic Review; healthy nutrition is affected by COVID-19 preventive measures (e.g., restrict physical contact). | Academia | 2021 | Not applicable  COVID-19 | Neira C, Godinho R, Rincón F, Mardones R, Pedroso J. Consequences of the COVID-19 Syndemic for Nutritional Health: A Systematic Review. Nutrients. 2021 Apr 1;13(4):1168. doi: 10.3390/nu13041168. PMID: 33916087; PMCID: PMC8065714. |
|  |  |  |  |  |
| 1. The syndemic of COVID-19 and dengue in areas for which dengue is endemic warrants further investigation to the syndemic nature of both diseases. | Academia, Hospital | 2021 | Peru  COVID-19 and dengue virus | Mejía-Parra JL, Aguilar-Martinez S, Fernández-Mogollón JL, Luna C, Bonilla-Aldana DK, Rodriguez-Morales AJ, Díaz-Vélez C. Characteristics of patients coinfected with Severe Acute Respiratory Syndrome Coronavirus 2 and dengue virus, Lambayeque, Peru, May-August 2020: A retrospective analysis. Travel Med Infect Dis. 2021 Sep-Oct;43:102132. doi: 10.1016/j.tmaid.2021.102132. Epub 2021 Jun 26. PMID: 34186213; PMCID: PMC8234266. |
|  |  |  |  |  |
| 1. Implement syndemic perspectives to address maternal disparities and prevention during the COVID-19 pandemic. | Academia | 2020 | United States  Maternal health and COVID-19 pandemic | Lemke MK, Brown KK. Syndemic Perspectives to Guide Black Maternal Health Research and Prevention During the COVID-19 Pandemic. Matern Child Health J. 2020 Sep;24(9):1093-1098. doi: 10.1007/s10995-020-02983-7. PMID: 32696248; PMCID: PMC7372977. |
|  |  |  |  |  |
| 1. The syndemic effect of the social determinants of health and COVID-19 on essential workers may present a challenge for which assuring equitable treatment and support for depression, anxiety, and support are important. | Academia, Hospital, Foundation | 2020 | Brazil and Spain  Depression, anxiety, lifestyle, and COVID-19 pandemic | De Boni RB, Balanzá-Martínez V, Mota JC, Cardoso TA, Ballester P, Atienza-Carbonell B, Bastos FI, Kapczinski F. Depression, Anxiety, and Lifestyle Among Essential Workers: A Web Survey From Brazil and Spain During the COVID-19 Pandemic. J Med Internet Res. 2020 Oct 30;22(10):e22835. doi: 10.2196/22835. PMID: 33038075; PMCID: PMC7641648. |
|  |  |  |  |  |
| 1. A syndemic framework is necessary to conceptualize the impact of SARS-CoV-2 among HIV patients and an urgent need to strengthen healthcare programs within Nigeria. | Academia | 2020 | Nigeria  SARS-CoV-2 and HIV | Akyala AI, Iwu CJ. Novel severe acute respiratory syndrome coronavirus 2 (SARS-CoV-2) co-infection with HIV: clinical case series analysis in North Central Nigeria. Pan Afr Med J. 2020 Sep 10;37:47. doi: 10.11604/pamj.2020.37.47.24200. PMID: 33209174; PMCID: PMC7648482. |
